# Agent-Based Modeling Demonstrates How Local Chemotactic Behavior Can Shape Biofilm Architecture

**DOI:** 10.1128/mSphere.00285-19

**Published:** 2019-05-29

**Authors:** Emily G. Sweeney, Andrew Nishida, Alexandra Weston, Maria S. Bañuelos, Kristin Potter, John Conery, Karen Guillemin

**Affiliations:** a Institute of Molecular Biology, University of Oregon, Eugene, Oregon, USA; b Institute of Ecology and Evolution, University of Oregon, Eugene, Oregon, USA; c College of Arts and Sciences Information Technology, University of Oregon, Eugene, Oregon, USA; d Humans and the Microbiome Program, CIFAR, Toronto, Ontario, Canada; University College Dublin, Belfield

**Keywords:** autoinducer 2, biofilms, chemotaxis, computer modeling

## Abstract

Bacteria are often found living in aggregated multicellular communities known as biofilms. Biofilms are three-dimensional structures that confer distinct physical and biological properties to the collective of cells living within them. We used agent-based modeling to explore whether local cellular interactions were sufficient to give rise to global structural features of biofilms. Specifically, we asked whether chemorepulsion from a self-produced quorum-sensing molecule, autoinducer-2 (AI-2), was sufficient to recapitulate biofilm growth and cellular organization observed for biofilms of Helicobacter pylori, a common bacterial resident of human stomachs. To carry out this modeling, we modified an existing platform, Individual-based Dynamics of Microbial Communities Simulator (iDynoMiCS), to incorporate three-dimensional chemotaxis, planktonic cells that could join or leave the biofilm structure, and cellular production of AI-2. We simulated biofilm growth of previously characterized H. pylori strains with various AI-2 production and sensing capacities. Using biologically plausible parameters, we were able to recapitulate both the variation in biofilm mass and cellular distributions observed with these strains. Specifically, the strains that were competent to chemotax away from AI-2 produced smaller and more heterogeneously spaced biofilms, whereas the AI-2 chemotaxis-defective strains produced larger and more homogeneously spaced biofilms. The model also provided new insights into the cellular demographics contributing to the biofilm patterning of each strain. Our analysis supports the idea that cellular interactions at small spatial and temporal scales are sufficient to give rise to larger-scale emergent properties of biofilms.

**IMPORTANCE** Most bacteria exist in aggregated, three-dimensional structures called biofilms. Although biofilms play important ecological roles in natural and engineered settings, they can also pose societal problems, for example, when they grow in plumbing systems or on medical implants. Understanding the processes that promote the growth and disassembly of biofilms could lead to better strategies to manage these structures. We had previously shown that Helicobacter pylori bacteria are repulsed by high concentrations of a self-produced molecule, AI-2, and that H. pylori mutants deficient in AI-2 sensing form larger and more homogeneously spaced biofilms. Here, we used computer simulations of biofilm formation to show that local H. pylori behavior of repulsion from high AI-2 could explain the overall architecture of H. pylori biofilms. Our findings demonstrate that it is possible to change global biofilm organization by manipulating local cell behaviors, which suggests that simple strategies targeting cells at local scales could be useful for controlling biofilms in industrial and medical settings.

## Abstract (precis)

Most bacteria exist in aggregated, three-dimensional structures called biofilms. Although biofilms play important ecological roles in natural and engineered settings, they can also pose societal problems, for example, when they grow in plumbing systems or on medical implants. Understanding the processes that promote the growth and disassembly of biofilms could lead to better strategies to manage these structures. We had previously shown that Helicobacter pylori bacteria are repulsed by high concentrations of a self-produced molecule, AI-2, and that H. pylori mutants deficient in AI-2 sensing form larger and more homogeneously spaced biofilms. Here, we used computer simulations of biofilm formation to show that local H. pylori behavior of repulsion from high AI-2 could explain the overall architecture of H. pylori biofilms. Our findings demonstrate that it is possible to change global biofilm organization by manipulating local cell behaviors, which suggests that simple strategies targeting cells at local scales could be useful for controlling biofilms in industrial and medical settings.

## INTRODUCTION

Bacteria often exist in aggregated, adherent communities called biofilms in which the cells are encased in a self-produced matrix. Bacteria residing in biofilms can exhibit resistance to environmental stressors such as antibiotics, changes in pH, and host immune defenses (1–3). Biofilms often adopt distinctive three-dimensional architectures with heterogeneous cell spacing that give rise to networks of channels. Biofilm architecture varies across bacteria, and while there is not yet a consensus on what function biofilm architecture plays, it has been shown that biofilm channels allow for the flow of oxygen, nutrients, and waste products into and out of the cell aggregates (2, 4–10). Biofilm architecture has also been implicated in conferring protection from bacteriophages, as well as being an important factor in mediating cooperative and competitive interactions among bacterial cells residing in the biofilm (11, 12). Biofilms play important ecological roles, ranging from supplying plant roots with nitrogen to removing biological matter from wastewater (2, 13–15). In certain contexts, biofilms create commercial and biomedical problems for society, from biofouling of municipal waterworks to life-threatening infections by pathogens harbored on medical implants or the lungs of cystic fibrosis patients (1, 16, 17). Therefore, being able to understand and ultimately manipulate biofilm assembly and disassembly would help address several major industrial, agricultural, and biomedical challenges through promoting assembly of beneficial biofilms and inhibiting formation of biofilms in harmful settings.

Biofilm assembly has been described alternatively as a developmental program controlled by stage-specific gene expression, similar to the development of a multicellular organism, or as the outcome of local adaptations of individual cells (3, 18–20). Distinguishing these two alternative possibilities is challenging because it can be difficult to discern whether biofilm phenotypes are achieved by optimizing group or individual fitness. For example, genes identified through forward genetic screens as being required for normal biofilm structures could be interpreted alternatively as being part of a biofilm genetic program or as controlling certain cellular behaviors that contribute to the self-assembly of biofilm structures (19, 20).

A distinguishing feature of the biofilm lifestyle is that cells in close proximity can produce and respond to secreted molecular signals on small spatial and temporal scales. One such example of a secreted signal is the class of quorum-sensing molecules that serve as density-dependent forms of communication to influence group behaviors. These can include molecules such as acylated homoserine lactones (AHLs) made by Gram-negative bacteria, the production of and response to which can vary considerably across different bacterial species. Another example of a quorum-sensing molecule is the tetrahydroxy furan molecule autoinducer-2 (AI-2), which is produced by many bacteria through a common metabolic pathway but elicits different responses through species-specific receptors. Quorum-sensing molecules often regulate gene expression, including genes involved in biofilm growth and dissolution, by acting through canonical signal transduction pathways (21–24). In this context, quorum-sensing molecules can be viewed as master regulators of biofilm developmental programs. AI-2 specifically has been shown to influence the overall structure of bacterial biofilms in diverse organisms such as *Bacillus*, *Streptococcus*, *Aggregatibacter, Pseudomonas, Escherichia, Vibrio* and *Helicobacter* (25–32). In addition to regulating gene expression, AI-2 can elicit more immediate and local behaviors in bacteria through chemotaxis signal transduction that directs bacterial movement relative to a chemical gradient (26, 33–37). In the case of Helicobacter pylori, we showed that AI-2 is perceived as a chemorepellent (38), whereas Escherichia coli perceives AI-2 as a chemoattractant (35).

Previous experimental work from our group showed that both H. pylori biofilm mass and structural patterning are influenced by AI-2 chemotaxis. To determine the role of AI-2 in H. pylori biofilm formation, we constructed strains that were defective for AI-2 production (*luxS* deleted), AI-2 chemoreception (*cheA, tlpB, aibA*, and *aibB* deleted), or overproduced AI-2 (*luxS* overproducer). We measured the biomass of the resulting biofilms using a crystal violet assay. We also measured the structural heterogeneity of the resulting biofilms by imaging them with fluorescence microscopy and quantifying a lacunarity metric that captures morphological features such as roughness of biofilm edges and patchiness of surface coverage. We observed that both AI-2 sensing and production mutants formed larger biofilms with more homogenous organization, whereas the strain that overproduced AI-2 formed smaller, more heterogeneously structured biofilms (26).

Our experimental observations are consistent with a role for AI-2 chemorepulsion in shaping biofilm structure. For example, bacterial cells that chemotax away from AI-2 would be motivated to leave or deterred from joining a biofilm that is a concentrated source of AI-2. Our experimental results, however, could not rule out the possibility that additional functions of AI-2 signaling, such as regulation of global gene expression programs, contribute to the overall architecture of H. pylori biofilms.

As a complement to experimental studies, computational modeling has played an important role in the study of biofilm assembly because it provides researchers with the opportunity to test and refine their understanding of the minimal set of parameters that can give rise to biofilm structures observed in nature (12, 39, 40). Agent-based models that treat each cell in the biofilm as independent agents are particularly well suited for exploring how individual cellular behaviors give rise to emergent properties of the biofilm. Here, we used agent-based modeling to ask whether individual cellular behaviors of AI-2 production and chemotaxis are sufficient to produce global features of biofilm structures observed experimentally.

For our agent-based modeling of AI-2 chemotaxis in biofilms, we used a well-established biofilm modeling platform, Individual-based Dynamics of Microbial Communities Simulator (iDynoMiCS) (41), which simulates behaviors of individual bacterial cells to understand larger, community behaviors. iDynoMiCS is an open-source platform and accessible for modifications and collaboration. We implemented several critical modifications to iDynoMiCS in order to explore whether AI-2 chemotactic responses could recapitulate our experimentally observed biofilm phenotypes. First, we expanded the models to include three-dimensional chemotaxis. We next included a population of planktonic (free-swimming) cells that were continually introduced into the bulk medium and could join the biofilm. Additionally, cells from the biofilm could leave and become part of the planktonic population. Finally, we introduced AI-2 as a chemorepellent compound that was produced by individual cells as a function of their metabolic capacity and that diffused through the three-dimensional space.

With the addition of AI-2 production and chemoreception to our modeling platform, we recapitulated our previous experimental data showing that biofilms of strains lacking the ability to produce or sense AI-2 were larger than wild-type (wt) biofilms. In addition, the architecture of the biofilms, including spacing of cell groups within the biofilms, matched well between the experimental and modeled biofilms. Finally, our modeling of biofilms contributed new insight into the demographics dictating biofilm size, suggesting that cell dispersal as a result of chemorepulsion is a major contributor to the reduced biofilm mass of AI-2-responsive versus nonresponsive cells. These results indicate the utility of our modified iDynoMiCS platform for studying chemotaxis in biofilm dynamics and provide support for the view that local cellular behaviors of AI-2 chemotaxis can explain global features of biofilm formation and patterning.

(This article was submitted to an online preprint archive [42].)

## RESULTS

### Addition of chemotaxis and AI-2 production to agent-based modeling of biofilm formation.

Agent-based models are useful tools for exploring how simple interactions between cells contribute to the overall properties of bacterial communities, such as biofilms. iDynoMiCS simulates biofilm formation by taking into account biologically relevant parameters, such as nutrient concentrations, nutrient diffusion rates, and cell division and spacing (see Table S1 in the supplemental material for parameters used in the simulation [41]). To investigate the role of AI-2-mediated chemotaxis in biofilm architecture, we extended the iDynoMiCS model by introducing several properties, including three-dimensional chemotaxis, planktonic (free-swimming) cells, cells joining and leaving the biofilm, and AI-2 production. These new iDynoMiCS additions were critical for exploring how chemoreception of AI-2 shapes H. pylori biofilms. In addition, these developments necessitated a new visualization tool that aided in data interpretation (see Materials and Methods).

10.1128/mSphere.00285-19.6 TABLE S1 Default iDynoMiCS parameters. Download Table S1, PDF file, 0.06 MB. Copyright © 2019 Sweeney et al. 2019 Sweeney et al. This content is distributed under the terms of the Creative Commons Attribution 4.0 International license.

**FIG 1:**
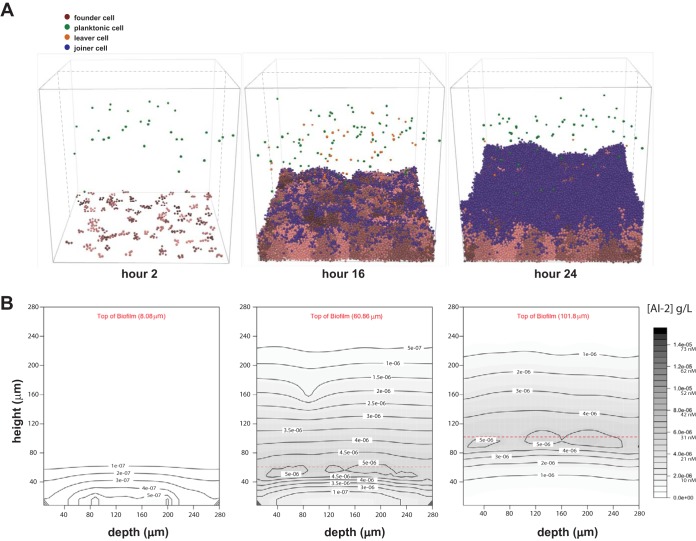
Time steps and AI-2 gradients of example wild-type iDynoMiCS-modeled H. pylori biofilm. (A) Wild-type biofilm after 2, 16, and 24 h of growth. Each sphere represents a modeled bacterial cell with colors corresponding to different cell behaviors. Note there is a mix of cells leaving, cells dividing from the original founding population, and cells joining the biofilm. Each grouping of pink cells represents a clonal population. (B) Shown are corresponding AI-2 concentration graphics below each time point shown in panel A. The AI-2 concentration is a representative vertical slice through the center of the three-dimensional modeled biofilm (gray dashed lines in panel A), with darker color representing higher concentrations of AI-2.

Our extended model starts with 100 bacterial cells randomly placed on the two-dimensional surface at the bottom of a container that is continually supplied with fresh, nutrient-containing medium. These cells expand and proliferate according to the iDynoMiCS growth and spacing algorithms. We allowed new planktonic cells (250 cells/h) to enter the container throughout each 24-h simulation (Fig. 1A and movies in the supplemental material). The planktonic cells moved through the space according to a chemotaxis algorithm (see Materials and Methods). Planktonic cells would join the simulated biofilm if they swam close enough to the biofilm surface and if the concentration of a chemorepellant was below the set chemoeffector threshold. In addition, cells at the biofilm edge could leave and enter into the planktonic pool. In our simulations, we chose the AI-2 chemoeffector threshold to be that at which planktonic cells contributed to the population of the biofilm to the extent that was defined as halfway between cells never joining the biofilm and cells always joining the biofilm (Fig. S1).

10.1128/mSphere.00285-19.1 FIG S1 Chemoeffector threshold paramaterization. Chemoeffector threshold values on the left represent more frequent negative chemotaxis responses (e.g., lower concentrations of AI-2 are required to elicit chemotaxis) in the system, and values on the right represent less frequent negative chemotaxis responses when all other parameters in the model were held constant. Each point represents the means and standard deviations from 10 to 25 replicate simulations. Download FIG S1, PDF file, 0.03 MB. Copyright © 2019 Sweeney et al. 2019 Sweeney et al. This content is distributed under the terms of the Creative Commons Attribution 4.0 International license.

After testing several models of AI-2 production, we chose a model that tied AI-2 production directly to the growth and metabolism of each cell or agent. This model is reasonable because AI-2 is produced as a by-product of the activated methyl cycle (24). We also incorporated into the model a constant cellular uptake of AI-2, which is common in bacteria (43). We do not yet know whether H. pylori has an active AI-2 uptake mechanism, but incorporating a constant uptake parameter best recapitulated our experimental measurements of AI-2 (26, 33). In the iDynoMiCS simulations, cells near the surface of the modeled biofilms have more access to fresh nutrients and therefore divide and produce AI-2 (environmental AI-2 concentrations shown in gray) at a higher rate than cells in the middle or bottom of the biofilm (Fig. 1B). The constant cellular uptake of AI-2 in the model resulted in a lower concentration of AI-2 in the volume at the bottom of the biofilm (Fig. 1B).

### Modeling recapitulates biofilm mass as a function of AI-2 chemorepulsion.

**FIG 2:**
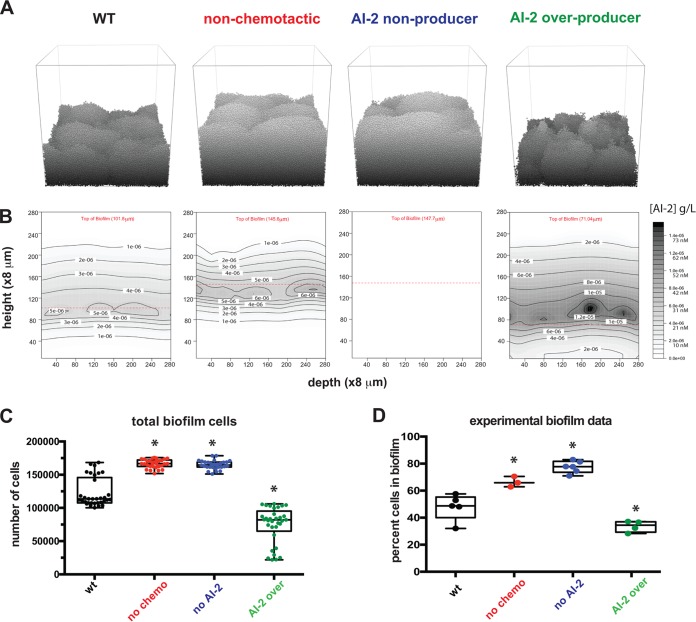
Modeling confirms AI-2 chemotaxis and production alter overall biofilm size. (A) Representative images of 24-h biofilms for each of the four strains in grayscale to show contours. To simplify, only the founding population, their progeny, and joiners are shown. Planktonic cells have been removed for simplicity. (B) The associated AI-2 gradients for panel A. (C) Total number of cells attached to the modeled biofilms at the 24-h time point (*n* = 30). (D) The sizes of the experimental biofilms from Anderson et al. (26) are graphed according to the percentage of cells in the biofilm (compared to planktonic cells). Asterisks indicate a significant difference from the wild type. Statistics for panels C and D were determined using a one-way analysis of variance (*P* < 0.05). Data in panel D are from Anderson et al. (26).

Using the model, we tested whether we could recapitulate the outcomes of our previous experiments demonstrating an important role for AI-2 production and chemorepulsion in H. pylori biofilm mass and patterning (26). To simulate these experiments, we modeled the strains and conditions used in this experimental work. The strains included wild-type cells (wt), cells unable to chemotax (no chemo), cells unable to produce AI-2 (no AI-2), and cells that overproduce AI-2 (AI-2 over). For each of these genotypes we ran 30 individual iterations and compared the number of cells in our simulated H. pylori biofilms to those of experimental work (Fig. 2). Wild-type cells produced moderately sized biofilms in both the model and the experimental setup, while cells that could not produce AI-2 or chemotax away from AI-2 produced larger biofilms. Finally, both experimental and modeling results revealed that the AI-2 overproducer made smaller biofilms. These data support our conclusion that our modeling platform could recapitulate experimental results.

### Modeling recapitulates the impact of AI-2 chemorepulsion on biofilm spatial organization.

**FIG 3:**
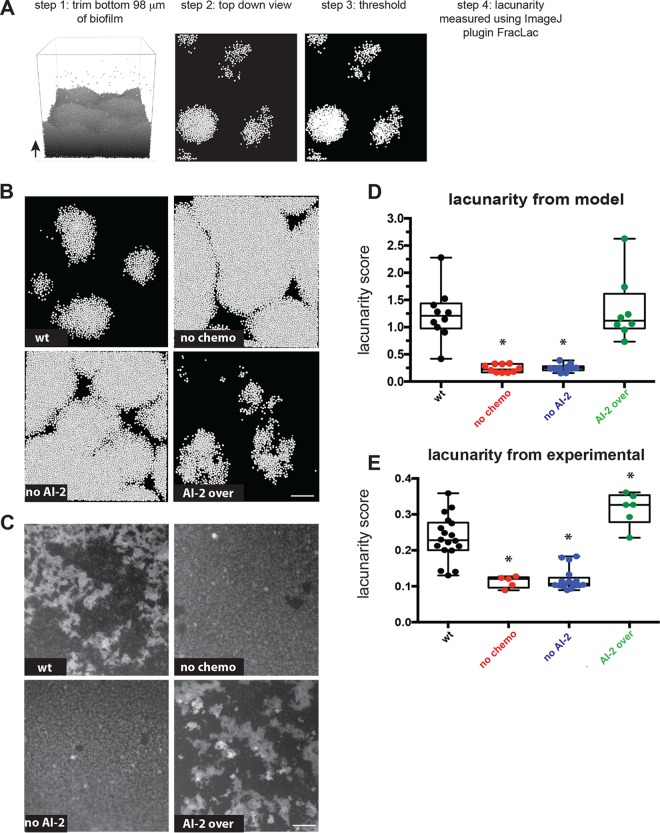
Modeling confirms AI-2 chemotaxis and production influence biofilm organization. (A) Lacunarity analysis pipeline for the modeled biofilm images. The bottom 98 µm was removed from each 24-h biofilm across all genotypes (see Materials and Methods). The top-down view is used to for comparisons to the experimental images in panel C. Using ImageJ, each image was thresholded and then analyzed with FracLac to determine the lacunarity score. More details can be found in Materials and Methods. (B) Example images of all four modeled genotypes from the top down. Bar, 40 μm. (C) Example images of experimental H. pylori biofilms grown on glass slides, from Anderson et al. (26). Bar, 100 μm. (D) Lacunarity scores graphed for modeled biofilms (*n* = 8 to 10). (E) Lacunarity scores graphed for each experimental biofilm for each genotype from Anderson et al. (26). Asterisks in panels D and E indicate a significant difference from the wild type. Results were determined using a one-way analysis of variance (*P* < 0.05). Data in panel E are from Anderson et al. (26).

We used modeling to confirm that AI-2 shapes H. pylori biofilm architecture. We had shown previously that we could quantify the heterogeneity of biofilms using a lacunarity metric, which measures spacing between patterns and boundary smoothness. Lacunarity measurements have been used across disciplines and size scales to compare not only how much space is filled by objects but also specifically how the space is filled (44, 45). To quantify space filling by biofilm cells using lacunarity, experimental biofilms were grown on glass slides, fixed, and stained with 4′,6-diamidino-2-phenylindole (DAPI) and visualized with epifluorescence. The cellular component of the biofilm was defined using an intensity threshold, and the resulting images were analyzed using an ImageJ plugin, FracLac, to quantify lacunarity (Fig. 3) (44–47). To compare these experimental results with our model outputs, we performed a similar analysis of the top layer of 10 simulated biofilms for each genotype (Fig. 3A). Visually, the simulated biofilm structures (Fig. 3B) resembled the experimental data (Fig. 3C). The wild-type and AI-2-overexpressing strains produced biofilms with marked spacing between cell patches, whereas the nonchemotactic and non-AI-2-producing strains formed much more homogeneous structures. Although the experimental and modeled biofilms were analyzed on different size scales, plotting the resulting lacunarity scores revealed a striking similarity between experimental and modeling data (Fig. 3D and E).

### Modeling predicts subcellular populations that contribute to biofilm mass.

**FIG 4:**
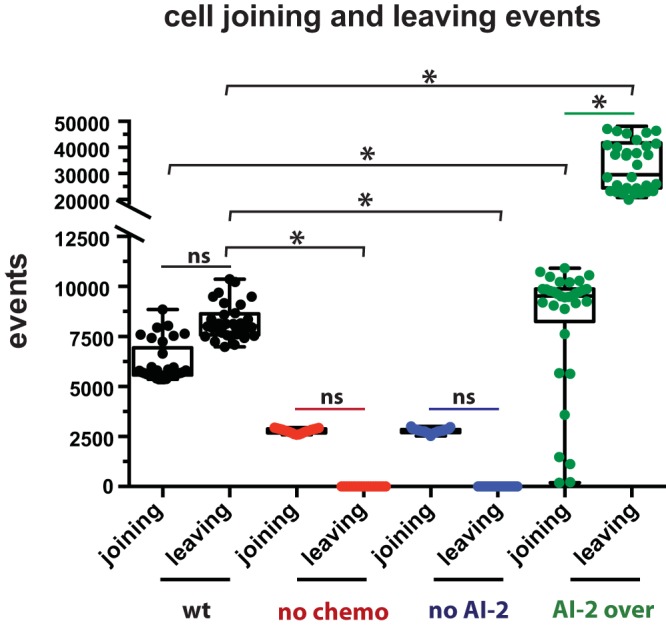
Modeling suggests that AI-2 chemotaxis and production influence biofilm cell demographics. Each leaving and joining event from 0 to 24 h of the modeled biofilms was graphed by genotype (*n* = 30). Asterisks indicate a significant difference; results were determined using a one-way analysis of variance (*P* < 0.05; ns, not significant).

The model afforded us the opportunity to examine the cellular demographics contributing to biofilm mass, which would be difficult to do experimentally. We modeled biofilm formation for 24 h in 30 parallel simulations and tallied the individual leaving events and joining events. Wild-type biofilms showed equivalent numbers of joining and leaving cells (Fig. 4). As expected, biofilms of nonchemotactic and non-AI-2-producing cells had no leaving events, since AI-2 chemorepulsion was the only leaving mechanism in the model. The AI-2 overproducer strain had a dramatic increase of leaving events, which was expected given the higher concentration of AI-2 near the surface of the biofilm (Fig. 2B), that would drive cells to chemotax away from the biofilm. Interestingly, both the nonchemotactic and non-AI-2 producers had a reduction in the number of joining events compared to the wild-type population despite not experiencing chemorepulsion from the biofilm. Also counterintuitively, there were more overall joining events in the AI-2 overproducer strain biofilm than in the wild-type population. As discussed below, the number of joiners could be explained by the differences in architectures and specifically surface areas and joining opportunities afforded by the growing biofilms of the different strains. Overall, our modeling supports the idea that AI-2 chemorepulsion promotes a balance of leaving and joining events that influences global biofilm growth.

## DISCUSSION

In this study, we used agent-based modeling to explore the extent to which local cell chemotactic responses to a self-produced molecule could explain biofilm growth and patterning properties. By extending the iDynoMiCS modeling platform to include three-dimensional chemotaxis, cell joining and leaving events, and AI-2 production, we were able to recapitulate our experimental observations of H. pylori biofilm formation with a collection of strains with different AI-2 production and perception properties (26). We showed that cells unable to make or chemotax away from AI-2 produced larger biofilms than wild-type cells. These biofilms also differed in their organization, with more homogenous cell spacing and smaller gaps between cell clusters. Overproduction of AI-2 resulted in smaller and more heterogeneously spaced biofilms. Biofilms are complex structures with towers and channels that facilitate fluid flow for efficient oxygen and nutrient permeation, waste excretion, and cell turnover (2, 4–6). We found that by modeling local chemotactic responses to a self-produced molecule, we could simulate the assembly of biofilms with the global property of high lacunarity, characteristic of biofilms with extensive channels (Fig. 3). The agreement between our simulations and experimental results supports the idea that local cellular behaviors, such as production and chemoavoidance of AI-2, can explain global architectural features of bacterial biofilms.

One notable discrepancy between the model and the experiments is that iDynoMiCS simulates biofilms under a constant supply of nutrients, whereas the experimental biofilms were grown under static conditions, as batch cultures (26). This discrepancy could account for certain differences between the modeled and experimental results. For example, the model simulated additional planktonic cells introduced from the bulk medium at each time step and resulted in greater biofilm mass over the 24-h period than observed experimentally. However, because our investigation focused on the effects of an endogenously produced cellular product that was supplied almost exclusively by the static biofilm as opposed to the bulk medium, we feel that the model has utility for understanding the role of AI-2 chemotaxis in biofilm growth.

Our modeling approach allowed us to dissect the demographics of biofilm assembly in a way that would be difficult to do experimentally without sophisticated genetic tools for marking cell lineages. As expected in our model, cells left the biofilm when they were programmed to chemotax away from AI-2, and they left in greater numbers when the biofilm cells produced more AI-2. We did not initially expect the wild-type and AI-2 overproducer populations to have more cells join the biofilms than the populations without chemotaxis or AI-2 production. However, inspection of the biofilm structures assembled in these different models showed that the wild-type and overproducer biofilms had many more gaps and edges, creating more extensive surface area that planktonic cells could stochastically encounter and then join at a certain probability. This difference in surface area and joining opportunities could explain the higher numbers of joiners in the populations of cells engaging in AI-2 chemorepulsion. In addition, the heterogeneous architectures of these biofilms would create local minima in AI-2 concentrations and opportunities for joining even in the context of AI-2 chemorepulsion. Differences in the local AI-2 concentration landscapes could explain the higher number of joiners seen with the AI-2 overproducer versus wild-type cell populations.

Biofilms can have detrimental impacts on humans when formed on medical devices or at sites of infection. Our modeling shows how structural features of biofilms, such as extensive surface area or channels, are affected by the concentration of endogenously produced molecules. In the case of H. pylori, endogenously produced AI-2 is a chemorepellent and promotes biofilm dispersal (26). Manipulation of AI-2 has been proposed as a strategy for managing pathogen infections, for example, to disperse deleterious biofilms (48). Our modeling, however, suggests that this strategy results in unanticipated consequences. For example, for bacteria that chemotax away from AI-2, like H. pylori, increasing AI-2 levels could create more biofilm surface area and result in increased incidence of new bacteria joining the biofilm. This would be especially problematic in a scenario of ecological succession in which antibiotic-resistant or more virulent strains were available to join.

Although our model recapitulated several features of AI-2-dependent biofilm assembly observed experimentally, it is based on certain assumptions about AI-2 fluxes that are likely to be oversimplifications. In our current model, AI-2 production is linked to metabolic activity and uptake is constant. When examined experimentally, parameters of AI-2 production, uptake, and sensing are known to vary greatly between bacterial species and depending on cells’ metabolic states (49–52). Using deterministic simulations of AI-2 production from a system of ordinary differential equations, Quan and colleagues showed that variability in AI-2 uptake within a modeled biofilm can lead to desynchronization of autoinduction across the community (53), highlighting the importance of considering heterogeneities in AI-2 fluxes. In addition, AI-2 could be produced from sources other than the bacterial constituents of a biofilm. For example, mammalian host tissues were recently shown to synthesize an AI-2 mimic that is sensed by bacterial AI-2 receptors (54). Future iterations of the model could incorporate more detailed parameters of AI-2 fluxes, but these would need to be tailored to the specific bacterial species and environments being modeled.

Most bacteria exist not in monocultures but rather in multispecies consortia (55). AI-2 is known to contribute to the organization of such consortia, for example, in biofilms that assemble on the enamel surfaces of teeth (56, 57). Recently, Laganenka and Sourjik showed that in a simple two-member biofilm community of Enterococcus faecalis and Escherichia coli cells, AI-2 chemotaxis plays an important role in biofilm growth and patterning. In this model community, both species produce AI-2 but only E. coli chemotaxes toward it (37). Importantly, the spatial arrangement of different species within a biofilm can strongly influence cooperative and competitive interactions occurring between neighboring cells, and this in turn can affect overall biofilm fitness and, in the case of pathogens, alter virulence (58). Therefore, an appealing direction for our work is to apply our modeling approach to multispecies communities composed of members with known AI-2 properties. By assigning simple AI-2 production, chemoattraction, and chemorepulsion behaviors to different members, one could explore the extent to which local AI-2 chemotactic responses could explain global spatial patterning observed in multispecies communities.

## MATERIALS AND METHODS

### Computational modeling of biofilms.

The simulation of the growth of biofilms was accomplished using the agent-based modeling package iDynoMiCS (version 1.1, January 2013). Individual cells are represented as discrete spherical agents with programmable behaviors that are subject to influence from other agents and their surrounding environments. The model consists of an evenly spaced grid of three dimensions with three regions: the biofilm (region I), the boundary (region II), and the bulk (region III) (41). The bulk compartment at the top represents well-mixed bulk solutes that interface with the bacterial compartment at the bottom through a diffusion boundary layer. Solutes are represented by concentration fields changing due to diffusion and from uptake by the cells in the bacterial compartment that provides a surface for initial seed cells to attach. As the cells take up solutes, they can grow and divide or die above or below certain set size thresholds. These processes of growth and division lead to mechanical stress between the cells, which is relieved through a shoving algorithm. This shoving and the simulation of other physical forces on the biofilm dictate the formation of the biofilm’s structure.

To represent a bacterial population with both biofilm-attached cells and planktonic cells and to simulate the dynamics of cells joining and leaving a biofilm, we extended iDynoMiCs to include new agents with attributes and behaviors specific to planktonic cells. This version is available at https://github.com/alexwweston/iDynoMiCS. Cells can be either biofilm associated or planktonic cells capable of movement in two or three dimensions. At each 1-h time step, a set number of planktonic cells are initially placed at random *x* and *z* coordinates just below the bulk compartment with an initial angle aimed downwards away from the bulk so that they do not immediately leave the system. Subsequently each individual planktonic cell will move at a random distance between 0 and its maximum distance at a random angle. All random numbers were generated from a uniform distribution. If a planktonic cell leaves the boundaries of the bacterial compartment, it is removed from the simulation. If a planktonic cell ends its movement within a certain distance from a biofilm-associated cell, it will then switch from planktonic to biofilm-associated behaviors. The maximum distance to move and the threshold distance for joining a biofilm are simulation parameters.

Planktonic cells are also given behaviors to simulate chemotaxis responses. Here, the goal was not to model how bacterial cells sense chemoeffectors (which involves detecting chemicals across a temporal gradient) but rather to model the consequence of chemosensing in terms of bacterial cell spatial distributions. In the model, a chemotaxing planktonic cell has attributes it identifies from the solute as a chemoeffector, whether or not it exhibits an attractive or repellent response to this chemoeffector, and the threshold for recognizing this chemoeffector, termed the chemoeffector threshold. Before moving, a planktonic cell will detect the concentration of its chemoeffector at its current location. If it is above its chemoeffector threshold, it will detect the gradient of the chemoeffector directly and move at an angle toward or away from this gradient depending on its response.

The attributes and behaviors of biofilm-associated cells are additionally extended to simulate biofilm-associated cells leaving the biofilm and becoming planktonic. A biofilm-associated cell has attributes for its chemoeffector, a threshold for recognizing this chemoeffector, or the chemoeffector threshold and a probability for leaving the biofilm if this threshold is surpassed. At the end of each interval in the simulation, cells on the periphery of the biofilm will check the local concentrations of their chemoeffector. If the concentration is above its chemoeffector threshold, that cell has a chance of leaving the biofilm at a frequency equal to its leaving probability. Upon leaving the biofilm, that cell becomes a planktonic cell and moves from the biofilm at a random angle away from the chemoeffector gradient. The chosen chemoeffector threshold (70 nM) corresponds to the concentration of AI-2 at which planktonic cells contribute to the population of the biofilm at the midpoint between cells never joining the biofilm and cells always joining the biofilm, with all other parameters held constant (see Fig. S1 in the supplemental material).

After trying several modes of AI-2 production, we chose to model AI-2 production as dependent on the growth reaction (e.g., AI-2 production is proportional to growth rate), with continuous uptake by the cells. AI-2 is produced only as cells grow, while uptake is constant, whether the cell is growing or not.

Diffusion parameters for AI-2 were also incorporated into the model. The parameters we used were the default diffusivity parameters of iDynoMiCS. In this model, diffusion and uptake of AI-2 occur at a much higher rate than the rate of cell growth; therefore, the assumption is that AI-2 concentration fields reach a pseudo-steady state, and at the end of each time step a steady-state solver is used (41, 59).

### Setting up and running simulations.

We simulated the movement of bacteria through a 280- by 280- by 280-µm space for a period of 24 h. The space was modeled as a 33 by 33 by 33 grid. Fluid movement was simulated using a major time step size of 1.0 h, and bacterial behaviors (movement, joining, and leaving) were updated at minor time steps of 0.05 h (3 min). These time steps allowed appropriate monitoring of the behaviors of the agents during the simulation (division, chemotaxis, joining, and leaving of the biofilm). Each simulation was seeded with 100 bacterial cells randomly placed on the bottom layer of the simulated grid, and 250 planktonic cells were added per hour from the bulk medium. Outputs for visualization were recorded at the end of every major time step. Other parameters for concentration and diffusivity of solutes and the cell attributes of agents were taken from measurements of E. coli biofilms used in other simulations under iDynoMiCS (41). Erosion and sloughing processes that can be modeled in iDynoMiCS were turned off for these simulations in order to focus on leaving events that are directly tied to AI-2. A full list of these parameters that were default iDynoMiCS parameters and were static in our simulations is provided in Table S1.

We tested a wide range of values for the new chemoeffector threshold parameter introduced in our model (Fig. S1). The value we chose of 70 nM AI-2 lies within a physiologically reasonable range between the minimum concentration of AI-2 that elicits dispersal of H. pylori biofilms experimentally (37 nM) and the maximum concentration of AI-2 measured in H. pylori cultures (0.1 mM) (26, 33, 38). Bacterial growth kinetics were modeled using the Monod growth equation with an additional term representing the production of AI-2. The values for these parameters and equations for the wild-type strain used in the simulations are found on Table S2. Mutant strains used in the simulations use minor modifications of these values, which are highlighted in Table S3. The mutant chemotaxis strain is given an infinite value for its chemoeffector threshold, causing it to never detect its chemoeffector. The mutant overproducer strain is given a larger AI-2 yield coefficient. Because we have observed experimentally that H. pylori *luxS* mutants have a growth rate similar to that of wild-type H. pylori (33), in our model we had the mutant strain defective in AI-2 production synthesize an arbitrary alternative product in its growth reaction, such that it experienced a biosynthetic burden similar to that of the wild-type strain.

10.1128/mSphere.00285-19.7 TABLE S2 Parameters added to iDynoMiCS. Download Table S2, PDF file, 0.06 MB. Copyright © 2019 Sweeney et al. 2019 Sweeney et al. This content is distributed under the terms of the Creative Commons Attribution 4.0 International license.

10.1128/mSphere.00285-19.8 TABLE S3 Parameters changed during simulations. Download Table S3, PDF file, 0.04 MB. Copyright © 2019 Sweeney et al. 2019 Sweeney et al. This content is distributed under the terms of the Creative Commons Attribution 4.0 International license.

### Visualizing the biofilms.

The visualization of the agent-based simulation of bacterial biofilms was created using custom-built codes developed in C++, using OpenGL for the graphics and Qt for the user interface. The simulations are run within iDynoMiCS, which exports the entirety of the simulation in XML. Each bacterium is displayed as a sphere that has a radius dictated by the simulation and a color based on the bacterial type and, in some scenarios, modified based on family, genealogy, generation, or birthday. Each founding cell is labeled in a different shade of pink, and the daughter cells remain the same color as the original founding cell to allow for recognition of clones. The code is open source and can be downloaded at https://bitbucket.org/kpotter/vizr.

The visualization of the AI-2 gradients via contours was done using the R library filled.contours. To create these images, the data are loaded into R, a single slice of the data volume is extracted at a specified time point, and these data are used as the input to the contours function. The R code is provided in the supplemental material.

### Calculating lacunarity.

The simulated biofilms grew more rapidly than the experimental biofilms, such that when viewed from the top, they appeared 100% confluent by 24 h; however, the spatial packing of the cells was not uniform. To compare the spatial organization of the simulated and experimental biofilms, we restricted our analysis to the top portion of the simulated biofilms corresponding to the volume with the most cell dynamics. To decide the extent of the bottom portion of the biofilm to exclude from our spatial analysis, we calculated the percentage of surface area covered by cells across all experimental wild-type images using ImageJ and determined this value to be approximately 43% percent coverage. We next determined that removing the bottom volume of height of 98 µm from the simulated biofilms resulted in 43% coverage for a representative set of wild-type biofilms, viewed from the top down (Fig. 3A). Therefore, the bottom volume of height of 98 µm was removed from all simulated biofilms and lacunarity was determined. To determine the lacunarity score, we opened the experimental or trimmed simulated biofilm images in ImageJ, converted them to grayscale, adjusted the threshold to a set cutoff to differentiate cells from noncells (black and white only), and analyzed the resultant images using the FracLac plugin (46).

### Data availability.

We have updated version 1.1 of iDynoMiCS to include several new parameters, including AI-2 production and uptake. The new associated source code and documentation for running the software is available in Weston and Nishida (60).

10.1128/mSphere.00285-19.2 MOVIE S1 Simulation of wt biofilm development. Download Movie S1, MOV file, 5.8 MB. Copyright © 2019 Sweeney et al. 2019 Sweeney et al. This content is distributed under the terms of the Creative Commons Attribution 4.0 International license.

10.1128/mSphere.00285-19.3 MOVIE S2 Simulation of nonchemotactic biofilm development. Download Movie S2, MOV file, 9.2 MB. Copyright © 2019 Sweeney et al. 2019 Sweeney et al. This content is distributed under the terms of the Creative Commons Attribution 4.0 International license.

10.1128/mSphere.00285-19.4 MOVIE S3 Simulation of AI-2 nonproducer biofilm development. Download Movie S3, MOV file, 9.8 MB. Copyright © 2019 Sweeney et al. 2019 Sweeney et al. This content is distributed under the terms of the Creative Commons Attribution 4.0 International license.

10.1128/mSphere.00285-19.5 MOVIE S4 Simulation of AI-2 overproducer biofilm development. Download Movie S4, MOV file, 6.7 MB. Copyright © 2019 Sweeney et al. 2019 Sweeney et al. This content is distributed under the terms of the Creative Commons Attribution 4.0 International license.

10.1128/mSphere.00285-19.9 TEXT S1 Supplemental materials and methods. Download Text S1, PDF file, 0.04 MB. Copyright © 2019 Sweeney et al. 2019 Sweeney et al. This content is distributed under the terms of the Creative Commons Attribution 4.0 International license.
