# Supplementary material for: Agent-Based Modeling Demonstrates How Local Chemotactic Behavior Can Shape Biofilm Architecture
Source: mSphere. 2019 May 29;4(3):e00285-19. doi: 10.1128/mSphere.00285-19 (PMC6541737; doi:10.1128/mSphere.00285-19)
Supplement: TABLE S1 [file mSphere.00285-19-st001.pdf]

Supplemental Table 1. Default iDynoMiCS parameters.

Parameter Name	Value	Description (if needed)
**Simulation Parameters**		
Output Period	1 hours	
Time Step Initial	1 hours	
End of Simulation	24 hours	
Agent Time Step	0.05 hours	3 min
Starting Solute Concentrations	0	
Nutrient Concentration in Bulk	10e-3 g/L	
Nutrient Diffusivity	1e-4 m^2/day	
AI-2 Diffusivity	2e-4 m^2/day	
Biomass Density	150 g/L	density of active biomass
Inert Density	150 g/L	density of inert biomass
Capsular Density	75 g/L	density of capsular material (EPS)
Grid Dimensions	33x33x33	264x264x264 µm
Size of Grid Unit	8 µm	
Boundary Layer	50 µm	thickness of the boundary layer between the biofilm and bulk
Biofilm Diffusivity	0.8	relative diffusivity of biofilm compared to water
Specific Area	80 m^2/m^3	surface to volume ratio of the reactor
Erosion	off	
Sloughing	off	
Shoving Max Nodes	2.00E+06	default parameters for iDynoMiCs shoving algorithm
Shoving Fraction	0.025	default parameters for iDynoMiCs shoving algorithm
Shoving Max Iterations	250	default parameters for iDynoMiCs shoving algorithm
Shoving Mutual	FALSE	default parameters for iDynoMiCs shoving algorithm
**Cell Parameters**		
Biomass/Inert Mass	0 fg	initial mass of each particle
Division Radius	2 µm	cell radius threshold for division
Division Radius CV	0.1 µm	coefficient of variation for division radius
Death Radius	0.2 µm	cell radius threshold for cell death
Death Radius CV	0.1 µm	coefficient of variation for death radius
Baby Mass Fraction	0.5	fraction of cell mass a baby cell receives
Baby Mass Fraction CV	0.05	coefficient of variation for baby mass fraction
Shove Factor	1.15	factor multiplying the cell's radius to give the shove radius, the radius used determine whether two cells overlap and need to be shoved
Shove Limit	0	minimal distance between two cell's shove radius needed to be maintained
EPS Max	0.1	maximum fraction of cell mass taken up by the EPS capsule before excretion of EPS
kHyd	0.007 / hr	hydrolysis rate, rate at which capsular EPS is excreted to neighboring EPS
Starting # of Randomly Seeded Cells	100 cells	100 cells/264 μm^2 = 0.38 cells/μm^2
