# Supplementary figures and images for: Agent-Based Modeling Demonstrates How Local Chemotactic Behavior Can Shape Biofilm Architecture
Source: mSphere. 2019 May 29;4(3):e00285-19. doi: 10.1128/mSphere.00285-19 (PMC6541737; doi:10.1128/mSphere.00285-19)

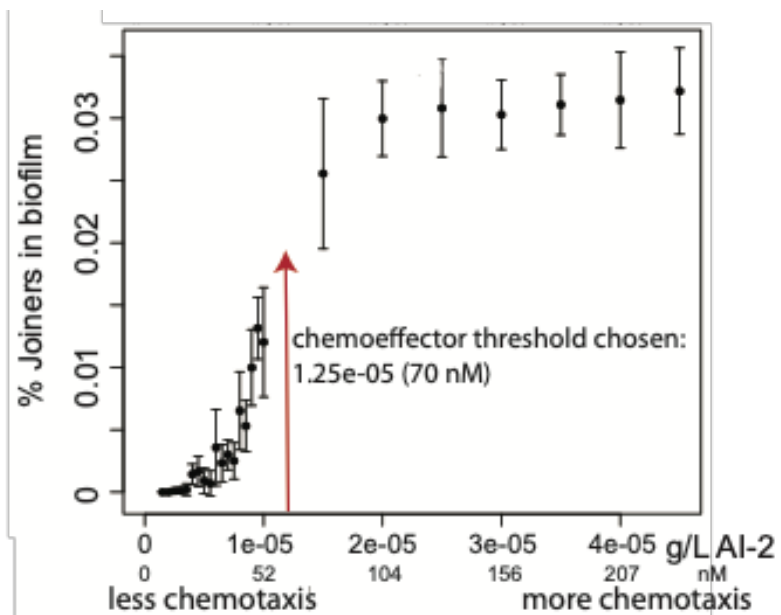

Supplement: FIG S1 [file mSphere.00285-19-sf001.pdf]
