# Supplementary material for: Agent-Based Modeling Demonstrates How Local Chemotactic Behavior Can Shape Biofilm Architecture
Source: mSphere. 2019 May 29;4(3):e00285-19. doi: 10.1128/mSphere.00285-19 (PMC6541737; doi:10.1128/mSphere.00285-19)
Supplement: TABLE S2 [file mSphere.00285-19-st002.pdf]

Supplemental Table 2. Parameters added to iDynoMiCS.

**Wildtype Parameter Values**		
**Parameter Name**	**Value**	**Description (if needed)**
# of Planktonics Entering per Time Step	250	
Planktonic Travel Distance	25 µm	maximum distance a planktonic cell can travel
Planktonic Attachment Distance	3 µm	minimum distance required for a planktonic cell to attach to a biofilm-associated cell
Planktonic Chemoeffector	AI-2	
Planktonic Chemoeffector Threshold	1.25e-5 g/L	70 nM
Planktonic Chemoeffector Response	repellent	
Biofilm-Associated Chemoeffector	AI-2	
Biofilm-Associated Chemoeffector Threshold	1.25e-5 g/L	70 nM
Biofilm-Associated Leaving Probability	0.05	probability of a biofilm-associated cell leaving if chemoeffector threshold exceeded
**Monod Kinetic Growth with AI-2 Reaction Parameters**		
Solute	AI-2	
Catalyzed by	biomass	
muMax	0.7 / hour	maximum specific rate at which the reaction may occur
Ks of Nutrients	3.5e-5 g/L	half-maximum concentration of nutrients
Nutrient Yield Coefficient	-1.5	
Biomass Yield Coefficient	1	
Solute Yield Coefficient	0.01	
**Monod Kinetic AI-2 Uptake Reaction Parameters**		
Solute	AI-2	
Catalyzed by	biomass	
muMax	0.7 / hour	maximum specific rate at which the reaction may occur
Ks of Solute	3.5e-5 g/L	half-maximum concentration of solute
Solute Yield Coefficient	-0.01	
