# Supplementary material for: Agent-Based Modeling Demonstrates How Local Chemotactic Behavior Can Shape Biofilm Architecture
Source: mSphere. 2019 May 29;4(3):e00285-19. doi: 10.1128/mSphere.00285-19 (PMC6541737; doi:10.1128/mSphere.00285-19)
Supplement: TABLE S3 [file mSphere.00285-19-st003.pdf]

Supplemental Table 3. Parameters changed during simulations.

Parameter	Value in WT	Value in No Chemo	Value in AI-2 Over	Value in No AI-2
Biofilm-Associated/Planktonic Chemoeffector Threshold	1.25e-5 g/L	infinity	1.25e-5 g/L	1.25e-5 g/L
Monod Kinetic Growth with AI-2 Solute Yield Coefficient	0.01	0.01	0.02	0.01
Monod Kinetic Growth with AI-2 Solute Produced	AI-2	AI-2	AI-2	AI-X
