# Supplementary material for: Agent-Based Modeling Demonstrates How Local Chemotactic Behavior Can Shape Biofilm Architecture
Source: mSphere. 2019 May 29;4(3):e00285-19. doi: 10.1128/mSphere.00285-19 (PMC6541737; doi:10.1128/mSphere.00285-19)
Supplement: TEXT S1 [file mSphere.00285-19-s0001.pdf]

Supplemental Material and Methods

Visualizing the biofilms

References:

iDynoMiCS - http://www.birmingham.ac.uk/generic/idynomics/index.aspx

Qt - https://www.qt.io

filled.contours - https://stat.ethz.ch/R-manual/R-devel/library/graphics/html/filled.contour.html

--- R Code ---

```
require(XML)

require(RColorBrewer)


## Variables for specifying the data file

wtBase1 <- "biofilm/wt/2_wt(20151101_2148)/"

timestep16<- "env_State/env_State(16).xml"


## Set the current data

base <- wtBase1

timestep <- timestep16

dataPath <- paste(base,timestep, sep="")

xmlData <- xmlParse(dataPath)


## Convert from xml list to data matrix

solutePath <- "//solute[@name='AI2']/text()"

solute <- xpathApply(xmlData, solutePath, xmlValue)

flatList <- strsplit( gsub("\n", "", unlist(solute)) ,";")[[1]]

dataMatrix <- array(as.numeric(flatList), c(35, 35, 35))


## Grab a slice

slice <- dataMatrix[16,,]


## Set the resolution and the number of levels

x <- 1:35

y <- 1:35
```

```
z <- 1:35
levels <- seq(0, 1.554e-05, length.out = 30)


## Define the colormap
gray <- function(x)rev(gray.colors(x, start = 0.0, end = 1.0))


## Contour the data
filled.contour(x,y,
        slice,
        plot.axes = {axis(1);
                axis(2);
                contour(x,y,slice,   nlevels = 8, add = T);
                #contour(x,y,slice,   nlevels = 3, add = T)
               },
        color = gray,
        levels=levels,
        plot.title = title(main = plotTitle, xlab = "Depth (*8 ums)", ylab = "Height (*8 ums)") )
```
